# Prenatal diagnosis of tracheo-oesophageal fistula/oesophageal atresia: is MRI helpful?

**DOI:** 10.1038/s41390-024-03503-x

**Published:** 2024-08-29

**Authors:** Louise Wilson, Elspeth H. Whitby

**Affiliations:** 1 School of Medicine and Population Health, University of Sheffield, Sheffield, UK; 2 Medical Imaging and Medical Physics, Sheffield Teaching Hospitals, Sheffield, UK

## Abstract

**Background:**

Oesophageal atresia (OA) with or without tracheo-oesophageal fistula (TOF) affects 2.75 per 10,000 births within the UK. It is most frequently suspected on antenatal imaging when the stomach is absent or appears small. Studies have shown fetal magnetic resonance imaging (MRI) has greater diagnostic accuracy than ultrasound; however, there remains uncertainty over what size constitutes a small stomach and how frequently this correlates with a diagnosis of TOF/OA.

**Methods:**

A retrospective study of patients referred for fetal MRI due to suspicions of TOF/OA on antenatal ultrasound from 2011 to 2022. We also included patients with a fetal MRI suspecting TOF/OA who had been referred for other reasons. The indication, MRI findings and postnatal outcome were compared to assess diagnostic accuracy. For each case, the size of the stomach bubble was measured on MRI, and stomach volumes in a control group were measured for comparison.

**Results:**

The positive predictive value for USS was 45.5% and 51.7% for fetal MRI. Fetal MRI had a negative predictive value and sensitivity of 100% (*p* = 0.027). The control group showed a strong positive correlation between stomach size and increasing gestational age (*R*^2^ = 0.69, *p* < 0.001), but this correlation was less positive in the TOF/OA group (*R*^2^ = 0.26, *p* = 0.03), and the stomach volumes in TOF/OA were consistently lower than the control group. The receiver operating characteristic curve illustrates that an absent stomach or unmeasurably small stomach is more diagnostic of TOF/OA as volumes ≤0.06 ml had 90% sensitivity.

**Conclusion:**

Fetal MRI can accurately exclude TOF/OA but only has marginally improved positive predictive value over ultrasound. Research with larger numbers is required to further aid the development of a cut-off value for what can be considered a pathologically small stomach.

**Impact:**

- There are several features on imaging that raise the suspicion of TOF/OA.
- Fetal MRI has some improved diagnostic accuracy compared with antenatal ultrasound alone; however, it is only marginally better.
- Absence of stomach bubble and presence of oesophageal dilatation combined on fetal MRI are more diagnostic of TOF/OA.

## Introduction

Oesophageal atresia (OA) with or without tracheo-oesophageal fistula (TOF) affects 2.75 per 10,000 births within the UK.^1^ It is commonly suspected on ultrasound scans (USS) where the stomach bubble is difficult to visualise as filling of the stomach with amniotic fluid during fetal swallowing is disrupted. Over recent years there has been increasing use of fetal magnetic resonance imaging (MRI) clinically to aid with diagnosis of congenital anomalies. The importance of early and accurate diagnosis in TOF/OA is vital to aid counselling for parents and planning for management after delivery.

**Fig. 1:**
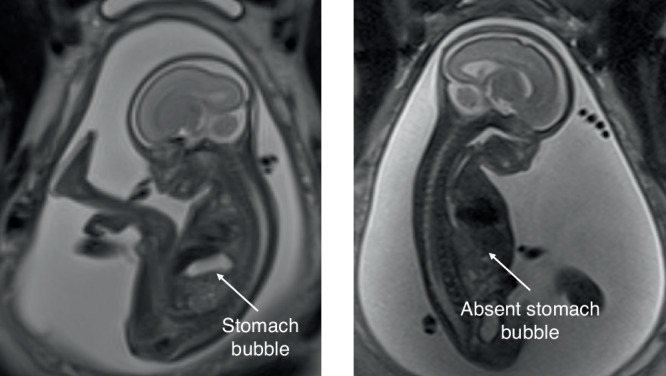
Comparison of normal and absent stomach bubble. Sagittal T2 SSFSE fetal MRI images of normal stomach bubble size in a 22-week fetus (left) in comparison with an absent stomach bubble in a 30-week fetus with TOF/OA (right).

A recent systematic review found that a small or absent stomach was identified in half of cases of OA, as shown in Fig. 1. This sign is subjective, however, with no consensus on what constitutes a “small” stomach.^2^ Research has shown that fetal stomach size increases with gestational age; this has been observed on both MRI and USS,^3–5^ and whilst a pilot study found that stomach size in fetuses with OA was smaller,^5^ there is no current cut-off value for normal. In addition, visualisation of the stomach bubble may occur intermittently in a healthy fetus due to the periodic nature of fetal swallowing. Furthermore, the presence of a distal tracheo-oesophageal fistula, which is seen in 85% of cases, means the stomach bubble may be seen on scans, and amniotic fluid volume may be normal.^6^ In cases of pure OA with no fistula, the stomach may still be visualised as a result of secretions produced by the gastric mucosa.^7^

**Fig. 2:**
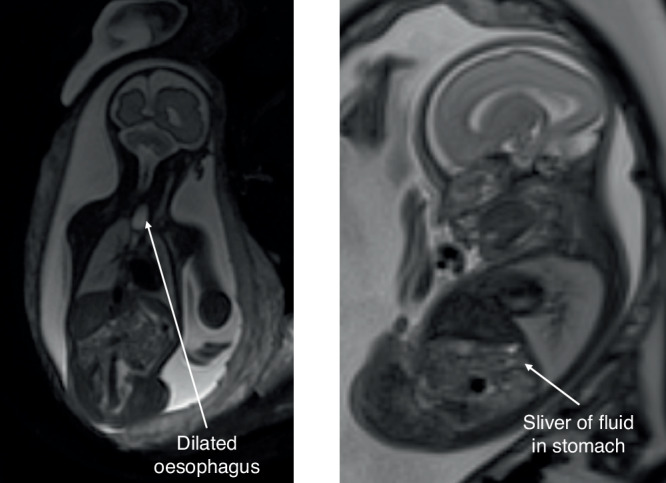
The pouch sign and a sliver of stomach fluid. Coronal T2 SSFSE MRI image of the dilated oesophagus (pouch sign) and small stomach in a 30-week fetus with TOF/OA (left) and sagittal T2 SSFSE fetal MRI image of a 25-week fetus with a sliver of fluid visible in the stomach (right).

Other features seen on imaging such as the “pouch sign”^8^ and the “distended fetal hypopharynx”^9^ have been proposed as a more reliable sign for diagnosis of OA especially when using fetal MRI.^10^ The pouch sign is a visualisation of the dilated blind-ending upper oesophagus seen in the neck or mediastinum during swallowing, as shown in Fig. 2. The pouch sign is seen most commonly in cases of pure oesophageal atresia, meaning MRI has an advantage in diagnosis of these cases. The distended fetal hypopharynx is seen as a result of amniotic fluid being forced upward into the mouth, distending the hypopharynx due to obstructed swallowing. Whilst these dynamic processes can be observed in real-time fetal sonography, the cine mode of fetal MRI allows for better dynamic studies. However, the pouch sign is not seen before 26 weeks of gestation^11^ and although the distended fetal hypopharynx has been identified at an earlier gestational age it was less specific for OA than the pouch sign (67% vs 97%).^9^

In cases of oesophageal atresia with tracheo-oesophageal atresia, the pouch sign and distended fetal hypopharynx are less likely to be observed on antenatal imaging; therefore, stomach size is a key diagnostic indicator.

The aims of this study were to determine the diagnostic accuracy of fetal MRI in comparison with antenatal ultrasound in TOF/OA and to review the stomach bubble size to see if a range of normal values could be obtained.

## Methods

The study had ethical approval from the Health Research Authority (HRA) as part of wider research into the use of fetal MRI in congenital anomalies of the fetal body (IRAS project ID 222053 and REC reference 17/EE/0162). As this is a study of diagnostic accuracy, the Standards for Reporting of Diagnostic Accuracy Studies (STARD) guidelines were consulted as a point of reference.^12^

**Fig. 3:**
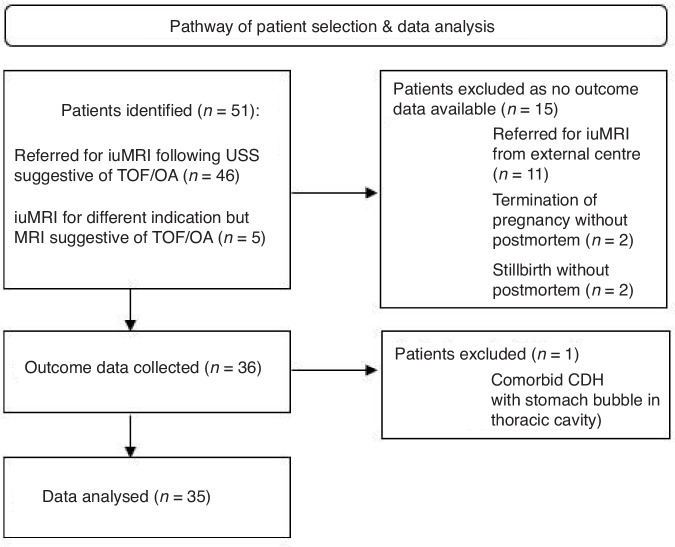
Patient selection and analysis. iuMRI in utero magnetic resonance imaging, USS ultrasound scan, TOF tracheo-oesophageal fistula, OA oesophageal atresia, CDH congenital diaphragmatic hernia.

This study was a retrospective review of all patients referred to our centre for a fetal MRI between October 2011 and October 2022 due to concerns regarding possible TOF/OA on USS. Common reasons for referral included polyhydramnios and a small or absent stomach bubble. We also included cases which had been referred for a fetal MRI for a different indication, but possible TOF/OA was suspected following the MRI scan. Inclusion criteria for the study were patients with features suggested of TOF/OA on USS referred for fetal MRI and patients with fetal MRI suggestive of TOF/OA who had been referred for other reasons. All antenatal ultrasounds were performed by consultant fetal medicine specialists with over 3 years of consultant experience. A flow chart of patient recruitment is shown in Fig. 3.

The MRI scans were performed using a 1.5 Tesla Siemens Avanto scanner (Erlangen, Germany). Stomach volume assessments were made from the T2 single-shot fast spin echo (SSFSE) images (echo train length 117, repetition time 90, echo time 1000, number of excitations 1, matrix size 192 × 192, field of view 288 × 288 mm, flip angle 150, slice thickness 4 mm no gap).

Outcome data with overall final postnatal diagnosis made by imaging, surgery or post-mortem examination was collected for all patients and used as the reference standard to determine the diagnostic accuracy of the imaging. For all cases, independent of the outcome, the MRI scans were reviewed, and stomach bubble size was measured or documented as absent or unmeasurable in cases where only a sliver was visible. Other features, such as the presence of oesophageal dilatation, absence of the lower oesophagus, intermittent filling of the stomach, and regurgitation of fluid during fetal swallowing using the cine mode, were also documented.

The fetal stomach volumes were traced on each slice in either the coronal or sagittal orientation (whichever it was most visible on) using the Agfa Healthcare (Mortsel, Belgium) Enterprise Imaging platform. The areas of each slice were then summed and multiplied by the MRI slice thickness to calculate the total stomach volume. The stomach volume measurements were undertaken by two researchers working independently and any discrepancies were then reviewed by the research team together. One of the researchers has many years of experience in reporting fetal MRI scans. Analysis of inter-observer variation and intra-observer variation was then undertaken using Cohen’s weighted Kappa coefficient.

A second cohort of control patients was identified from fetal MRI scans which were undertaken for assessment of the placenta. These were healthy patients with no conditions which could affect stomach size. The stomach volumes for each of these patients were calculated using the method as described above, they were then plotted against gestation and compared with the stomach volumes in the cohort diagnosed with TOF/OA after delivery.

## Results

### Study characteristics

This study included a total of 51 patients; 46 of these were referred to our centre for fetal MRI due to concerns regarding TOF/OA on the ultrasound. The remaining five patients were referred for a fetal MRI for different reasons, but the possibility of TOF/OA was raised following the MRI. The pathway of patient selection and follow-up is summarised in Fig. 3.

The reasons for referral for fetal MRI to rule out TOF/OA (*n* = 46) included polyhydramnios (*n* = 12), a small/partially filled stomach (*n* = 15), an absent stomach (*n* = 18) and other associated anomalies (*n* = 1) which were other features of VACTERL association. The indications for MRI referral in the other five patients were congenital diaphragmatic hernia (CDH), spina bifida, dextrocardia, Dandy-Walker malformation and a single kidney in each patient, respectively.

The mean gestational age at the time of fetal MRI was 28.3 weeks. Outcome data with a final diagnosis was available for 36 patients. The remaining 15 patients with no outcome data of diagnosis were either referred from external centres (*n* = 11) or underwent termination of pregnancy (*n* = 2) or stillbirth (*n* = 2) but declined post-mortem examination. One patient was excluded from the analysis as they had a congenital diaphragmatic hernia with the stomach herniated into the thoracic cavity, which was felt to affect the reliability of stomach volume assessments. The patient characteristics and their MRI findings are summarised in Table 1.

**Table 1:** Patient characteristics and fetal MRI findings of patients with outcome data (*n* = 36).

	TOF/OA (*n* = 16)	No TOF/OA (*n* = 20)
Mean gestation at iuMRI	28.4 weeks	30.1 weeks
Reason for iuMRI		
Polyhydramnios	5	5
Small stomach	0	10
Absent stomach	10	5
Associated anomalies	0	1
Other condition suspected	3	0
iuMRI findings		
Small stomach	7	12
Absent stomach	5	2
Dilated oesophagus/pouch	4	1
Lower oesophagus not seen	3	1
Mean gestation at birth	36.6 weeks	38.1 weeks

Some patients had more than one finding.

*iuMRI* in utero magnetic resonance imaging, *TOF* tracheo-oesophageal fistula, *OA* oesophageal atresia.

The 35 patients with outcome data who were included in the analysis were all liveborn, with one death in the neonatal period in the confirmed TOF/OA group and three deaths from other causes in the patients without TOF/OA.

### Diagnostic accuracy of USS and MRI

For the patients with outcome data TOF/OA was suspected on USS in 33/35 cases and on fetal MRI in 29/35 cases. As discussed above, the differences in numbers are due to four patients being referred for fetal MRI due to other suspected diagnoses but no  suspicions of TOF/OA on ultrasound and because six MRI scans were reported as normal.

**Fig. 4:**
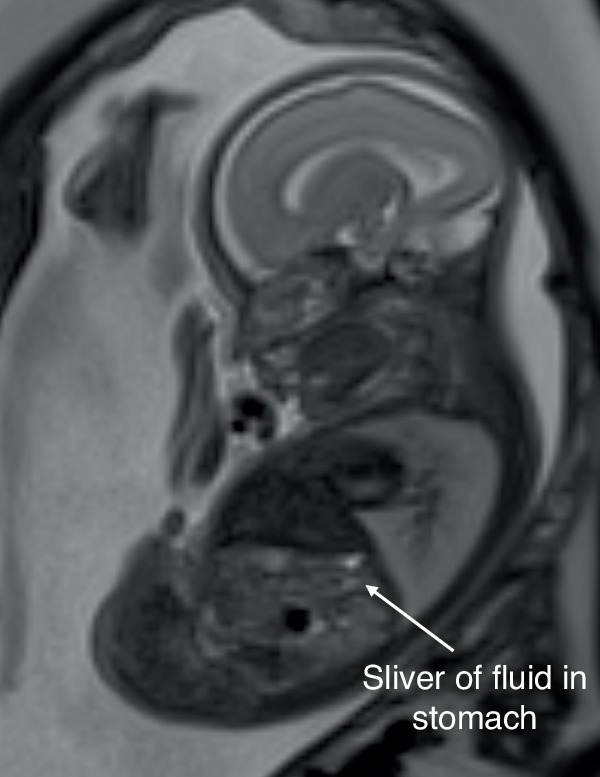
Sliver of stomach fluid. Sagittal T2 SSFSE fetal MRI image of a 25-week fetus with a sliver of fluid visible in the stomach.

The MRI findings suspicious for TOF/OA (*n* = 29) included a small/underfilled stomach (*n* = 16), an absent stomach bubble (*n* = 6), a sliver of fluid in the stomach (*n* = 5), intermittent filling of the stomach (*n* = 1), evidence of oesophageal obstruction/dilatation (*n* = 4) or absence of the lower oesophagus (*n* = 4). There was some overlap between findings in cases. As there is no recognised cut-off value for what determines a ‘small’ stomach, this finding was based on the opinion of the reporting radiologist, who has many years of experience with fetal MRI. This was recognised as a study limitation due to its subjectivity, however it reflects clinical practice and there is no objective definition of a small stomach at present. A sliver of fluid was defined as cases where the stomach was visible, but the area was too small to reliably measure on the MRI, as shown in Fig. 4. Attempted measurements of the stomach volumes where only a sliver was visible were undertaken, and these were all <0.2 ml; however, they were reported as unmeasurable as volumes were not felt to be reliable at this size.

Following delivery, there were 15 cases of confirmed TOF/OA of which seven had pure OA, seven had TOF/OA and one had a complete laryngo-tracheo-oesophageal cleft. Fetal MRI findings for these patients are shown in Table 2.

**Table 2:** iuMRI findings in confirmed patients by diagnosis.

	OA (*n* = 7)	TOF/OA (*n* = 8)	Complete cleft (*n* = 1)
Absent stomach	3	1	1
Small stomach	2	5	0
Oesophageal obstruction/dilatation	2	2	0
Lower oesophagus not visualised	3	0	0

Overlap in cases—two patients had a small stomach and lower oesophagus not seen; one patient had a small stomach and oesophageal dilatation.

*iuMRI* in utero magnetic resonance imaging, *TOF* tracheo-oesophageal fistula, *OA* oesophageal atresia.

Five patients had other associated congenital anomalies comprised of duodenal atresia (*n* = 1), cardiac abnormalities comprised of a ventricular septal defect (*n* = 1), cardiac dextroposition (*n* = 1) and Tetralogy of Fallot (*n* = 1) and VACTERL (Vertebral defects, Anal atresia, Cardiac defects, Tracheo-oesophageal fistula/oesophageal atresia, Renal abnormalities, Limb Abnormalities) association (*n* = 10). Of these 15 confirmed cases, 13 underwent MRI due to USS concerns regarding TOF/OA, but the other two were referred for fetal MRI for a different indication. The other indications for fetal MRI, which had confirmed TOF/OA, were spina bifida and dextrocardia on antenatal ultrasound.

The overall diagnostic accuracy for USS was 45.5% (15/33) and 51.7% (15/29) for fetal MRI. In this study, fetal MRI had a positive predictive value (PPV) of 51.7% but a negative predictive value (NPV) and sensitivity of 100% as all six MRI scans which were reported as normal did not have TOF/OA, i.e., there were no false negative results as shown in Table 3. These results were shown to be statistically significant, with a *p*-value of 0.027 using Fisher’s exact test. The diagnostic value of specific fetal MRI findings is highlighted in Table 4, and although certain findings, such as an absent stomach and oesophageal pouch, had a specificity of 95%, none of these findings were statistically significant when analysed in isolation.

**Table 3:** Diagnostic accuracy of fetal MRI.

	Sensitivity	Specificity	PPV	NPV	
Diagnosis TOF/OA	100%	30%	53.3%	100%	*p* = 0.024

*p*-value < 0.05 is considered statistically significant.

*TOF/OA* tracheo-oesophageal fistula/oesophageal atresia, *PPV* positive predictive value, *NPV* negative predictive value.

**Table 4:** Diagnostic value of individual fetal MRI findings.

	Sensitivity	Specificity	PPV	NPV	*p*-value
Small stomach	37.5%	40%	33.3%	44.4%	*p* = 0.18
Absent stomach	31.2%	95%	83.3%	63.3%	*p* = 0.07
Intermittent filling of the stomach	6.25%	100%	100%	57.1%	*p* = 0.44
Dilated oesophagus/pouch	25%	95%	80%	61.3%	*p* = 0.15
Lower oesophagus not seen	18.8%	95%	75%	59.4%	*p* = 0.30

*p*-value < 0.05 is considered statistically significant.

*PPV* positive predictive value, *NPV* negative predictive value.

As this study was based on a cohort of patients identified through the use of fetal MRI, the diagnosis was suspected on at least one imaging modality (ultrasound or MRI) prior to delivery. Further unpublished work was undertaken looking at all patients diagnosed with TOF/OA after birth who were managed at our centre from 2011 to 2023. A total of 48 patients were diagnosed with TOF/OA postnatally; however, only 33.3% were suspected on antenatal ultrasound and therefore referred for fetal MRI. 25% of this cohort were born in external district general hospitals. This highlights the importance of defining diagnostic criteria for TOF/OA to improve antenatal diagnosis.

### Stomach volume measurements

Stomach volumes measured from the cohort with outcome data (*n* = 35) ranged from 0.085 to 6.04 ml with a mean volume of 2.23 ml (*n* = 22) or were absent (*n* = 6) or a sliver of stomach was visible, but it was unmeasurable (*n* = 7). As previously discussed, measurements of these ‘slivers’ of fluid were made, but they were all <0.2 ml, which was felt to be unreliable at such small volumes. For the patients noted to have a visually normal stomach with no other features suspicious for TOF/OA on the MRI report, the measured volumes were 2.24–6.04 ml (mean volume = 4.02 ml). As discussed above, all of the patients with normal fetal MRI scans did not have TOF/OA. Of the 15 patients with confirmed TOF/OA, an absent stomach bubble was seen on MRI in five (83.3% of all absent stomach bubbles on MRI, i.e., 5/6), and 75% of patients with oesophageal obstruction/dilatation had TOF/OA confirmed after birth (3/4).

In the patients with confirmed TOF/OA after delivery (*n* = 15), the stomach bubble on MRI was absent in five and unmeasurable in four. In the six patients in whom the volume could be measured, it ranged from 0.349 to 3.55 ml, with a mean volume of 1.47 ml.

**Fig. 5:**
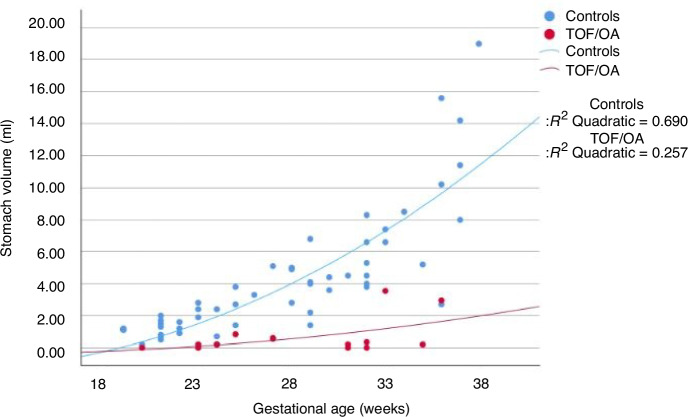
Scatterplot comparing the stomach size of controls and TOF/OA patients with increasing gestation.

The stomach volumes from the control patients (*n* = 51) were plotted against gestation and showed a positive correlation between stomach size and advancing gestation (*R*^2^ = 0.69) which was found to be statistically significant (*p* < 0.001) using the Pearson correlation coefficient. The stomach volumes for the patients with TOF/OA confirmed after delivery (*n* = 15) were then plotted on the same chart for comparison with the control group. Of these patients, only six had a stomach volume, which could be reliably measured; as for the remaining nine patients, five had an absent stomach, and four had only a sliver of fluid visible. Where the stomach bubble was absent, this was plotted as zero, and where only a sliver was visible, this was plotted as 0.5 ml. These results are summarised in Fig. 5 and show there is a less positive correlation between stomach volume and gestation in patients with TOF/OA (*R*^2^ = 0.257, *p* = 0.03) and that the stomach volumes in TOF/OA were consistently lower than the control group.

**Fig. 6:**
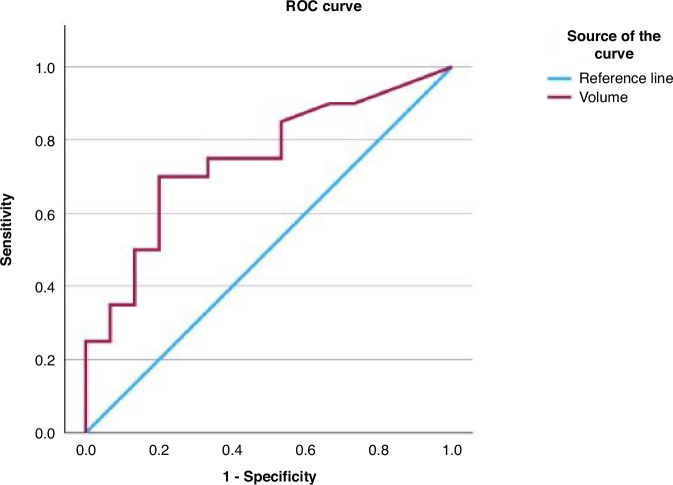
ROC curve of stomach volume, area under the curve = 0.747.

A receiver operating characteristic (ROC) curve was generated to see if a cut-off volume for stomach size indicative of TOF/OA could be determined. This data is limited by small numbers but shows an area under the curve of 0.75, as shown in Fig. 6. It shows that an absent stomach or unmeasurably small stomach (sliver) is more diagnostic of TOF/OA as volumes ≤0.06 ml had 90% sensitivity and 67% specificity.

An assessment of inter-observer variation and intra-observer variation was undertaken using Cohen’s weighted Kappa coefficient. The weighted Kappa was 0.818 for inter-observer variation and 0.883 for intra-observer variation, both of which showed excellent agreement.

## Discussion

Accurate and early diagnosis of TOF/OA is vital for perinatal counselling for families and appropriate planning for place of delivery and surgical management. There are multiple signs cited in the literature seen on both ultrasound and MRI which raise suspicion of TOF/OA but larger studies of the diagnostic accuracy of these signs on fetal MRI is lacking.^8^

This study has shown that fetal MRI has improved diagnostic accuracy over antenatal ultrasound alone, as seen in previous studies.^2^ In addition, it highlights the use of fetal MRI to accurately exclude TOF/OA with 100% sensitivity and negative predictive value which was statistically significant (*p* < 0.05). Evaluation of individual MRI findings has shown that absent stomach, dilated oesophagus and inability to visualise the lower oesophagus are highly diagnostic of TOF/OA with specificity of 95%. This is higher than in previous studies^13^ and most useful when these findings are seen in combination, although the specificity of these signs was not statistically significant when reviewed separately (*p* > 0.05).

However, the overall diagnostic accuracy of fetal MRI compared with ultrasound was only marginally better in this study (51.7% for MRI compared with 45.5% for USS). This is lower than previous studies which suggest MRI is much more accurate at correctly diagnosing TOF/OA antenatally.^2^ Therefore, this study raises the question of whether the additional costs and stress for the patient can be justified for only a marginal improvement in diagnostic accuracy.

The use of control data to show increasing stomach size with gestational age has only been published in one previous study using fetal MRI to conduct the measurements.^5^ Our data is consistent with what has previously been reported that there is a positive correlation between stomach volume and increasing gestation (*R*^2^ = 0.69). The comparison of these controls with a cohort of 15 patients with TOF/OA supports the evidence that stomach size in TOF/OA is consistently smaller than in the control group, and the stomach size has a less positive correlation with advancing gestation (*R*^2^ = 0.26).

It seems that stomach size alone is unlikely to be the only determinant of diagnosis, as we have shown multiple factors are involved and cases that are more complex, i.e., those with an absent stomach in addition to oesophageal dilatation or non-visualisation, have a much higher likelihood of TOF/OA. The ROC curve further confirms that an absent or unmeasurably small stomach on fetal MRI is indicative of pathology; however, a measurable but visually small stomach is less reliable in the diagnosis of TOF/OA.

In this cohort, there was only one patient with the combined features of absent stomach and oesophageal dilatation who did not have TOF/OA but was diagnosed postnatally with a complex upper airway anomaly (CHAOS syndrome). In addition to this, one of the patients in the cohort who had a visually small stomach on MRI, which was too small to be measured, was postnatally diagnosed with a congenital myopathy, and although TOF/OA could not be ruled out based on the MRI findings, there was a family history of congenital myopathy meaning this was felt to be the most likely diagnosis antenatally.

**Fig. 7:**
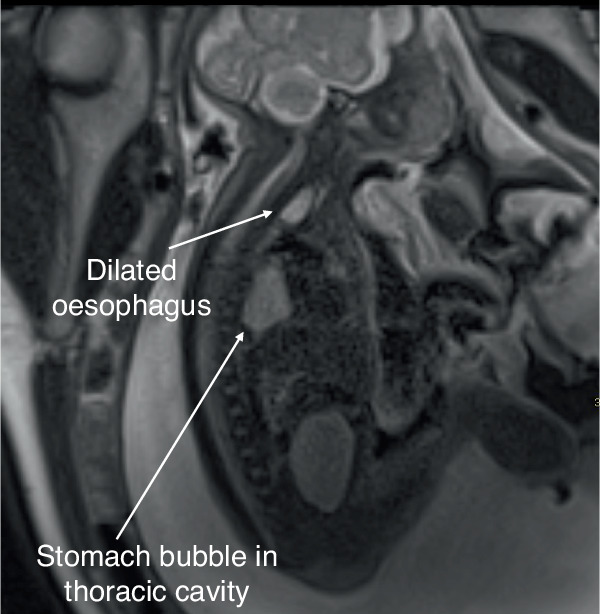
Comorbid TOF/OA and congenital diaphragmatic hernia (CDH). Sagittal T2 SSFSE fetal MRI image of a 35-week fetus with CDH and TOF/OA with rotated stomach bubble in the thoracic cavity and oesophageal pouch.

It is important to discuss the patient who was excluded from the study prior to analysis due to the presence of a left-sided congenital diaphragmatic hernia with the stomach bubble visible within the thorax. There was a concern that this may have affected the measurement of stomach volume. This patient was diagnosed postnatally with TOF/OA. Their stomach volume was a significant outlier when compared with the data set with a volume of 12.45 ml. For this patient, a bolus was seen in the oesophagus, raising the suspicion of TOF/OA from the fetal MRI, as shown in Fig. 7.

This study also highlights the prevalence of comorbidities associated with TOF/OA, such as cardiac abnormalities, congenital diaphragmatic hernia, VACTERL association and duodenal atresia. Research has shown that 55% of patients with TOF/OA have associated anomalies forming the VACTERL spectrum,^14^ including cardiac anomalies in 29%, gastrointestinal anomalies such as anorectal malformation in 16%, renal anomalies in 16% and musculoskeletal anomalies in 13%. The outcomes for these patients have been shown to be impacted by the presence of comorbidities, with rates of termination of pregnancy ranging from 3 to 8% in isolated TOF/OA^15^ but rising to 27% in the presence of other congenital anomalies.^16^ Survival rates have also been shown to be impacted by comorbidities, with rates of survival >90% in isolated cases and up to 87% in high-risk cases such as where there are cardiac anomalies. The role of fetal MRI in these cases is vital for comprehensive diagnosis to enable appropriate discussions with parents and within the wider multidisciplinary team.

This study is limited by sample size, which is further impacted by missing outcome data regarding the final diagnosis for 15/51 patients. As previously discussed, many of the signs suggestive of TOF/OA, such as a small stomach, are subjective, meaning there are no specific definitions in the literature. However, the lack of definition of a pathologically small stomach was one of the areas this study aimed to address. As a measurable stomach volume was seen in only six of the confirmed TOF/OA patients a reliable cut-off for a pathologically small stomach could not be determined by this data. The analysis using a ROC curve demonstrated that stomach volumes ≤0.06 ml, i.e., where there is an absent stomach or unmeasurable sliver of stomach seen on fetal MRI, have a sensitivity of 90%.

## Conclusion

In conclusion, this study has shown that fetal MRI has some improved diagnostic accuracy over antenatal ultrasound alone in the diagnosis of TOF/OA. It has a higher diagnostic specificity when findings such as an absent stomach bubble, oesophageal dilatation and non-visualisation of the lower oesophagus are present, especially when in combination. However, this improvement in accurate diagnosis is only marginal, which raises the question of whether a fetal MRI scan for suspected TOF/OA can be justified both in terms of the cost and stress for the family. This study shows that fetal MRI is accurate at ruling out TOF/OA as all MRIs reported as normal were confirmed as normal after delivery, meaning there were no false negative results.

## Data Availability

The datasets generated during and/or analysed during the current study are available from the corresponding author upon reasonable request.
